# Acute lymphoblastic leukemia in a 2-year-old girl whose mother was previously diagnosed with antiphospholipid syndrome: a case report

**DOI:** 10.1186/s13104-015-1104-1

**Published:** 2015-04-15

**Authors:** Miguel Ángel Castro-Jiménez, Carlos Efraín Cortés-Sánchez, Ernesto Rueda-Arenas, Lucy Adela Tibaduiza-Buitrago

**Affiliations:** Grupo Colombiano de Estudios Alfa en Epidemiología, Salud Poblacional, Estadística Aplicada y Ciencias Aliadas. Magna Science Corporation, Bogotá, D.C., Colombia; Grupo de Investigación Epidemiológica del Cáncer, Instituto Nacional de Cancerología, Avenida Calle 1 No. 9-85, Bogotá, D.C., Colombia; Postgrado en Salud Ocupacional. Universidad El Bosque, Avenida Carrera 9 No. 131 A – 02, Edificio Fundadores, Bogotá, D.C., Colombia; Departamento de Pediatría, Facultad de Salud. Universidad Industrial de Santander - Hospital Universitario de Santander, Carrera 33 No. 20-126., Bucaramanga, (Santander) Colombia

**Keywords:** Acute lymphoblastic leukemia, Child, Case report, Antiphospholipid syndrome, Maternal exposure

## Abstract

**Background:**

The role of maternal exposures and conditions in the origin of childhood cancer has been a subject of growing interest, but current evidence is inconclusive.

**Case presentation:**

We present a case detected in a multicenter case–control study evaluating the association between parental risk factors and childhood acute lymphoblastic leukemia (ALL). The patient is a Colombian girl who was diagnosed with ALL-L1 when she was 2 years old. Her mother had been diagnosed with antiphospholipid syndrome before pregnancy and had also been treated with subcutaneous injections of heparin. Other potentially relevant maternal and patient exposures are also reported in this paper.

**Conclusion:**

We hypothesize that the maternal autoimmune disease could be a contributor in the causality network of the daughter’s leukemia. However, the role of other exposures cannot be excluded.

## Background

This case was detected in a multicenter case–control study evaluating the association between parental risk factors and childhood acute lymphoblastic leukemia (ALL) [1,2]. In Western countries, ALL is the most common malignant disease in children under the age of 15 [3]. The etiologic factors contributing to the development of ALL have not been completely established, but include exposure to benzene, ionizing radiation, and some genetic syndromes [4]. Over the last few decades, interest has grown in the effect that certain parental exposures before birth or conception might have on the risk of childhood ALL [5-10]. Several environmental exposures and clinical conditions have been studied as hypotheses, but the results of these studies have been inconsistent [11-20]. Additionally, some authors have recently explored genetic variants and their role in the early stages of this type of leukemia [21-23]. Addressing the association between uncommon maternal disorders, such as some immunological diseases, and ALL in the offspring, which also has a low incidence, presents difficulties because of the low number of possible comparisons. We present a case of ALL in a girl whose mother had been previously diagnosed with a chronic immunological disease and discuss other potentially associated maternal and child exposures during three time periods: the last 24 months prior to the estimated date of conception of the patient, prenatal growth, and childhood development.

## Case presentation

The patient is a Colombian girl who was diagnosed at the age of 2 with the precursor B (common) form of ALL-L1 (CD10+, CD13+) with a normal karyotype. No molecular analysis was performed. The patient’s mother had been diagnosed with antiphospholipid syndrome (APS) and treated with subcutaneous injections of heparin (of unknown periodicity) since she was 26. The mother was 30 years old at the child’s birth and her blood type was A positive. She had undergone three spontaneous abortions before giving birth to her only live child (the case patient) and had been diagnosed with venous thrombosis when she was 16.

In the last 24 months before conception, the child’s mother was exposed to diagnostic X-rays in her abdominal region three times and took medications such as acetaminophen and nonsteroid anti-inflammatory drugs (NSAIDs). During the pregnancy there were various high-risk conditions such as threatened abortion, fetal distress, gestational diabetes, premature rupture of the membranes, and preterm labor. The mother took the prescribed prenatal multivitamin, supplemental ferrous pills, and acetaminophen daily. She also took a NSAID 1 day per month during the first 4 months of pregnancy and received a subcutaneous injection of heparin twice daily throughout the pregnancy.

The mother was not actively exposed to cigarette smoke, alcohol, or psychoactive drugs during the preconception or prenatal periods. However, she was exposed to passive smoking 7 days a week (less than five cigarettes per day) before the pregnancy.

The patient was born by cesarean section. Her weight, height, and blood type were 2240 g, 47 cm, and AB positive, respectively. At birth she was diagnosed with neonatal jaundice and unspecified problems with breathing. She was breastfed for 18 months. Prior to being diagnosed with ALL, the case patient presented with septic arthritis and was hospitalized. During this period she received several types of antibiotics (unspecified) and possibly other medications. She had been exposed to diagnostic X-rays seven times before the diagnosis of ALL for assessment of her thorax (n = 3), abdomen (n = 2), and right leg (n = 3). The patient was a passive smoker from birth until her diagnosis of ALL (<5 cigarettes per day/7 days per week). During all study periods (i.e., preconception, index pregnancy, and postnatal life), the case patient and/or her mother were continuously exposed to chemical products including thinner, gasoline, paints, and solvents because there was a woodworking shop in the building where they lived. At the time of the mother’s interview the patient was alive.

## Conclusions

Some parental exposures have been associated with an increased risk of childhood ALL [1,7,14]. In this report we have presented a case that is possibly related to a chronic maternal condition with alteration of immunological functions, its long-term treatment, or both. Previous studies have shown an association between childhood cancer and autoimmune disease in the patient [24] and between childhood cancer and a history of immunological disease such as APS in the parent [25-27]. Both APS and childhood ALL are diseases with low incidence rates in the general population. In fact, APS is estimated to have an incidence of approximately five new cases and a prevalence of 40 cases per 100,000 persons [28]. Therefore, studying the effects of uncommon maternal diseases on the risk of childhood ALL in children who were “exposed” to them, even prior to conception, represents a problem with respect to etiological evaluation because of the lack of an adequate sample size in terms of statistical power to confirm or discard any association.

We hypothesized that maternal APS might be related to the onset of ALL in this patient, although the causative role of other maternal and child exposures such as medications, passive smoking, and hydrocarbons (and other chemical products) cannot be excluded. Regarding the exposure to several X-rays, we need to take into account that this procedure was performed for the radiologic diagnosis or follow-up of recurrent infections that could be the first complications of an undiagnosed leukemia. Thus, it is possible that at least some of the X-rays were ordered after the actual onset of the leukemia, reducing the probability of a causal role in this case. Further epidemiologic studies designed to assess the relationship between maternal disorders such as APS and childhood ALL are needed.

## Consent

Written informed consent was obtained from the patient’s parents for publication of this Case Report and any accompanying images. A copy of the written consent is available for review by the Editor-in-Chief of this journal. This Case Report is based on a patient of a Colombian case–control study previously approved by the Industrial University of Santander Ethical Committee, the Javeriana University Ethical Committee, and the Institutional Review Boards of the participating hospitals.

## Abbreviations

ALL: Acute lymphoblastic leukemia
NSAID: Nonsteroid anti-inflammatory drug
APS: Antiphospholipid syndrome
